# Sofosbuvir-based regimen for genotype 2 HCV infected patients in Taiwan: A real world experience

**DOI:** 10.1371/journal.pone.0227424

**Published:** 2020-01-10

**Authors:** Wei-Lun Tsai, Chih-Feng Wang, Jin-Shiung Cheng, Wen-Chi Chen, Ming-Jong Bair, Ching-Chu Lo

**Affiliations:** 1 Division of Gastroenterology and Hepatology, Department of Internal Medicine, Kaohsiung Veterans General Hospital, Kaohsiung, Taiwan; 2 School of Medicine, National Yang-Ming University, Taipei, Taiwan; 3 Division of Gastroenterology and Hepatology, Department of Internal Medicine, Taitung Mackay Memorial Hospital, Taitung, Taiwan; 4 Mackay Medical College, New Taipei City, Taiwan; 5 Division of Gastroenterology and Hepatology, Department of Internal Medicine, St. Martin De Porres Hospital, Chiayi, Taiwan; 6 Chung-Jen junior College of Nursing, Health Sciences and Management, Chiayi, Taiwan; Harvard Medical School, UNITED STATES

## Abstract

**Background:**

Sofosbuvir (SOF)-based regimens achieve excellent efficacy and safety in the treatment of chronic hepatitis C (CHC) with various genotypes. There are few real-world instances of the use of SOF-based regimens to treat genotype 2 CHC. This study determines the effectiveness and safety of SOF/Ribavirn (RBV), SOF/Daclatasvir (DCV) and SOF/DCV/RBV in the treatment of genotype 2 CHC patients in Taiwan.

**Material and methods:**

Patients with genotype 2 CHC were treated for 12 weeks with SOF/RBV, SOF/DCV or SOF/DCV/RBV under the National Health Insurance reimbursement program in three hospitals in Taiwan. The sustained virological response at 12 weeks (SVR12) was determined. Adverse events were recorded for a safety analysis.

**Results:**

A total of 467 genotype 2 CHC patients were enrolled from January to October 2018. One hundred and eleven patients (24%) had cirrhosis, including 10 patients (2.1%) with hepatic decompensation. Fifty-five patients (12%) had already experienced interferon-alpha/RBV treatment. Forty-two patients (9%) had a history of hepatocellular carcinoma (HCC) in the baseline. Three hundred and fifty-five patients received SOF/RBV, forty-seven patients received SOF/DCV and sixty-two patients received SOF/DCV/RBV. The SOF/DCV group featured a greater HCV viral load than the SOF/RBV or SOF/DCV/RBV groups. SVR12 was achieved in 94.6% of the SOF/RBV group, 95.7% of the SOF/DCV group and 96.8% of then SOF/DCV/RBV group (P = NS). Thirteen out of 352 patients (3.7%) in the SOF/RBV group, 1 out of 62 patients (1.6%) in the SOF/DCV/RBV group and 1 out of 47 patients (2.1%) in the SOF/DCV group developed virological failure. There are no differences in virological failure between the three groups (P = NS). Multi-variate analysis shows that history of HCC is an independent factor that is associated with the failure of treatment in the SOF/RBV group (odds ratio:4.905, 95% confidence interval (CI): 1.321–18.205, P = 0.017). Hemoglobin levels at 12 weeks are significantly lower in the SOF/RBV and the SOF/RBV/DCV group than in the SOF/DCV group (P<0.05). Serious adverse events (SAE) occurred in six patients (1.6%) in the SOF/RBV group and in one patient (1.6%) in the SOF/RBV/DCV group. No patients in the SOF/DCV group experienced SAE.

**Conclusions:**

SOF/RBV, SOF/DCV or SOF/DCV/RBV for 12 weeks all achieve very high SVR rates and are equally effective in the treatment of genotype 2 CHC patients in the real world in Taiwan. Patients in the SOF/RBV group who have a history of HCC exhibit a lower SVR rate.

## Introduction

In Taiwan, hepatitis C virus (HCV) infection has a prevalence of around 2–5% and HCV is a major cause of liver cirrhosis and hepatocellular carcinoma (HCC) in Taiwan [1]. In patients with acute HCV infection, 60–90% become chronically infected with HCV (CHC) and after 20–30 years of infection, 20–30% develop cirrhosis of the liver or HCC [2,3]. Recent years, there have been significant progress in anti-HCV therapy. The resolution of the three-dimensional structures of several HCV proteins and the development of replicative cell culture systems has led to the identification of a number of potential targets for direct-acting antiviral (DAA) agents [4–5]. DAAs are very effective in the treatment of HCV and are associated with a significant decrease in liver-related morbidity and mortality [6–10]. Sofosbuvir (SOF) is an oral nucleotide analogue inhibitor of the NS5B polymerase of HCV. Phase 3 studies and real world data show that a combination of SOF and ribavirin (RBV) for 12 weeks produces a rate of sustained virological response (SVR) of 83–97% for genotype 2 CHC patients [11–19]. Other real world data show that SVR rates are lower but the independent predictor for the failure of treatment is rarely identified. Daclatasvir (DCV) is an inhibitor of NS5A of HCV. Several recent studies, DCV have added to SOF for the treatment of genotype 2 CHC with a SVR rate of 90–100% [20,21,22]. However, it is not clear that adding DCV to SOF with or without RBV increases SVR rates for the treatment of genotype 2 CHC. This study determines the effectiveness and safety of SOF/RBV, SOF/DCV and SOF/DCV/RBV for the treatment of genotype 2 chronic HCV patients in the real world in Taiwan.

## Patients and methods

### Study design

Since January 2017, the National Health Insurance Administration (NHIA) of Taiwan provides reimbursement for SOF-based DAAs for patients with advanced fibrosis who are infected with HCV. From January 2018 to October 2018, consecutive patients with genotype 2 chronic HCV who had advanced liver fibrosis and who received SOF/RBV, SOF/DCV or SOF/DCV/RBV were enrolled in a program that was sponsored by the NHIA of Taiwan in three hospitals (Kaohsiung Veterans General Hospital in southern Taiwan, St. Martin De Porres Hospital in central Taiwan and Taitung MacKay Memorial Hospital in eastern Taiwan). This is not a randomized study and in the clinical settings, patients were treated with SOF/RBV, SOF/DCV or SOF/DCV/RBV irrespective of the clinical condition. Advanced fibrosis was at least stage III liver fibrosis and was defined in terms of the presence of any one of the following: transient elastography (TE) with a liver stiffness measurement (LSM) ≥ 9.5Kpa [23], a Fibrosis-4 (FIB-4) score ≥ 3.25 [24], a liver biopsy presenting a METAVIR fibrosis score ≥ 3, [25] or ultrasound-diagnosis of liver cirrhosis with splenomegaly or gastroesophageal varices by endoscopy [26]. Key exclusion criteria included: active HCC before treatment, a Glomerular filtration rate (GFR) of less than 30 ml/min or an absolute contraindication for the use of SOF, DCV or RBV.

### Assessment and end-points

All enrolled patients received HCV RNA at baseline, 12 weeks on-treatment and 12 weeks end-of-treatment (EOT) and liver function tests, prothrombin time, renal function tests and complete blood counts at baseline and every 4 weeks during treatment and 12 weeks after EOT. Patients in the SOF/RBV group received SOF 400 mg once daily and, weight-based ribavirin (1000 or 1200 mg) twice daily. Patients in the SOF/DCV or SOF/DCV/RBV group received SOF 400 mg once daily and DCV 60 mg once daily with or without weight-based ribavirin (1000 or 1200 mg) twice daily. Anemia that developed during treatment was managed by reducing the RBV dose by 200 mg. Liver decompensation was defined as a Child–Turcotte–Pugh (CTP) classification of B or C. The presence of HCC was confirmed by a histological or image evaluation based on the recommendations of the current guidelines [27–29]. The primary efficacy endpoint was defined as the achievement of SVR12, which was defined as a HCV RNA level of less than the lower limit for quantification (LLOQ, 25 IU/ml) 12 weeks after stopping DAAs. All AEs were recorded and assessed according to the Common Terminology Criteria for Adverse Events (CTCAE) (version4.0).

### Statistical analysis

Clinical characteristics and clinical data were described using frequency counts and percentages for categorical variables and means with standard deviations for continuous variables. Group comparisons were performed using a Mann-Whitney Utest and a Pearson chi-square or a Fisher exact test for continuous and categorical variables, respectively. Covariates in the multivariable model were chosen *a priori* in terms of clinical importance. Each *p*-value is two-sided and is considered statistically significant if the *p*-value is less than 0.05. All analyses were performed using SPSS version 18.0.

### Ethics statement

This study was approved by the Institutional Review Boards of Kaohsiung Veterans General Hospital, St. Martin De Porres Hospital and Taitung Mackay Memorial Hospital. This is a retrospective study that does not involve intervention or obtaining clinical specimens and all the data is analyzed anonymously, so the need for informed consent was waived by the Institutional Review Boards of Kaohsiung Veterans General Hospital, St. Martin De Porres Hospital and Taitung Mackay Memorial Hospital. The patients’ medical records were accessed since January 2018 to April 2019.

## Results

### Characteristics of patients

A total of 341 patients who were chronically infected with genotype 2 CHC were enrolled from January to Aug 2019. 358 patients received SOF/RBV, 62 patients received SOF/RBV/DCV and 47 patients received SOF/DCV treatment. The demographic and baseline characteristics for the SOF/RBV, SOF/DCV/RBV and SOF/DCV groups are shown in Table 1. HCV RNA in the SOF/DCV group is higher than that for the SOF/RBV (P = 0.003) and SOF/RBV/DCV groups (P < 0.001). Total bilirubin level is higher in the SOF/DCV than the SOV/RBV group (P = 0.048). Age, sex, aspartate aminotransferase (AST), alanine aminotransferase (ALT), creatinine, hemoglobin, platelet, prothrombin, compensated or decompensated cirrhosis, treatment experienced and history of HCC are comparable for the SOF/RBV, SOF/RBV/DCV and SOF/DCV groups.

**Table 1:** Demographic characteristics of patients by treatment regimen.

	SOF+ RBV N = 358	SOF+RBV+DCV N = 62	SOF+DCV N = 47
Age (years)	64+11	63+13	65+12
Sex (M)	164 (46%)	14 (35%)	21 (47%)
HCV RNA (log10 IU/mL)	5.7+1**	5.5+1.0#	6.2+0.8#**
Albumin (g/L)	4.0+0.4	4+0.5	4.0+0.4
ALT (U/L)	80+67	78+79	63+59
AST (U/L)	67+48	64+48	54+37
Total Bilirubin (mg/dL)	0.8+0.4*	0.9+0.4	1.0+0.4*
Creatinine (mg/dL)	0.9+0.9	1.0+0.4	0.8+0.2
Hemoglobin (g/dL)	14+1.5	14+2.2	14+1.7
Platelet count (x109/L)	163+62	155+63	160+52
Prothrombin time	11.2+5.6	11+0.9	11+0.6
Cirrhosis Compensated Decompensated	83 (23%) 74 (21%) 9 (2.5%)	18 (29%) 17 (27%) 1 (2%)	10 (21%) 10 (21%) 0 (0%)
Treatment experienced	39 (11%)	9 (16%)	7 (15%)
HCC history	33 (9%)	5 (8%)	4 (9%)

# P<0.001

*P = 0.048

** P = 0.003

SOF: sofosbuvir, RBV: ribavirin, DCV: daclatasvir

### Efficacy

**Fig 1:**
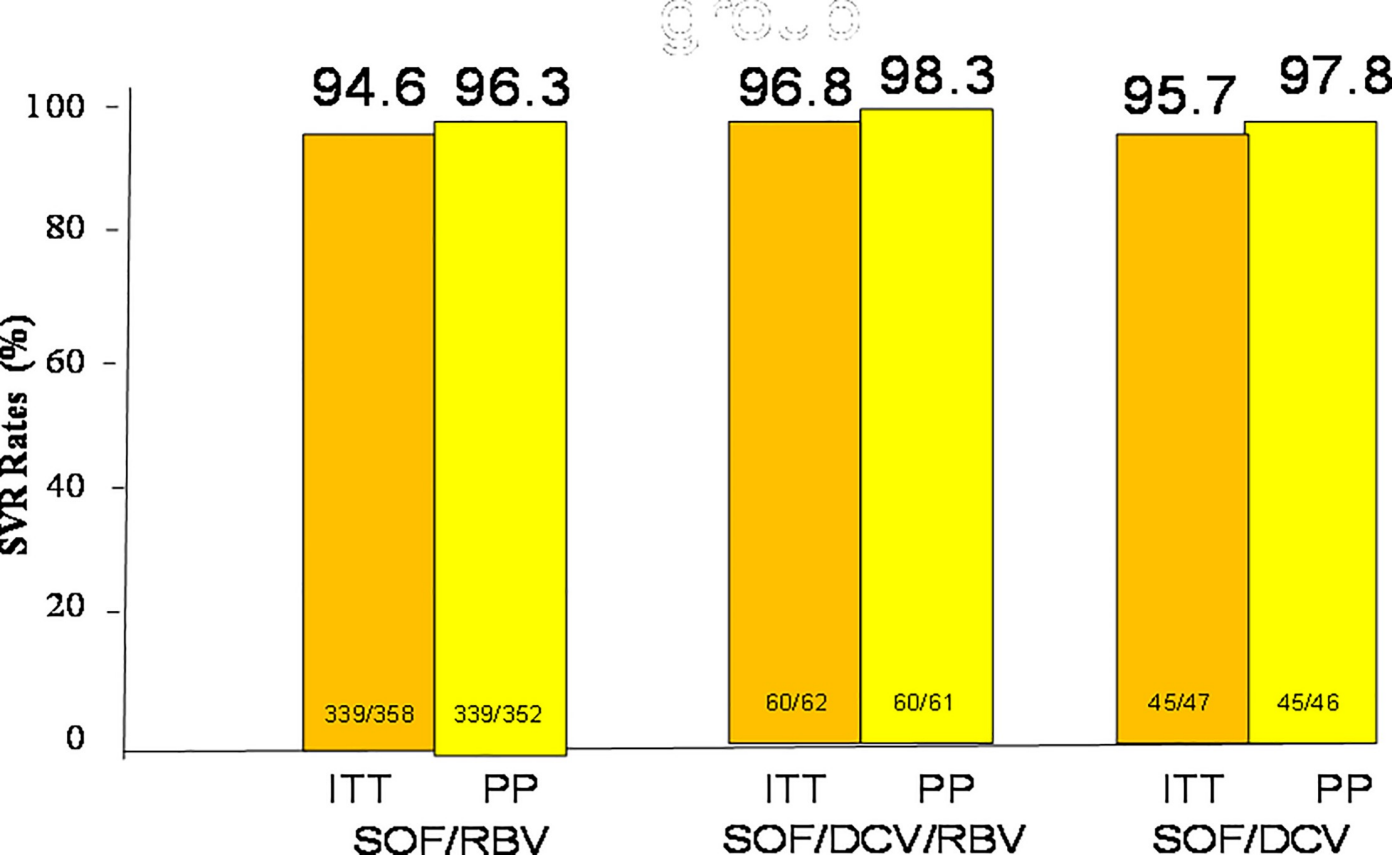
Sustained virologocal response (SVR) rates in patients who received SOF/RBV, SOF/DCV or SOF/DCV/RBV regimens. SOF: sofosbuvir, RBV: ribavirin, DCV: daclatasvir.

**Fig 2:**
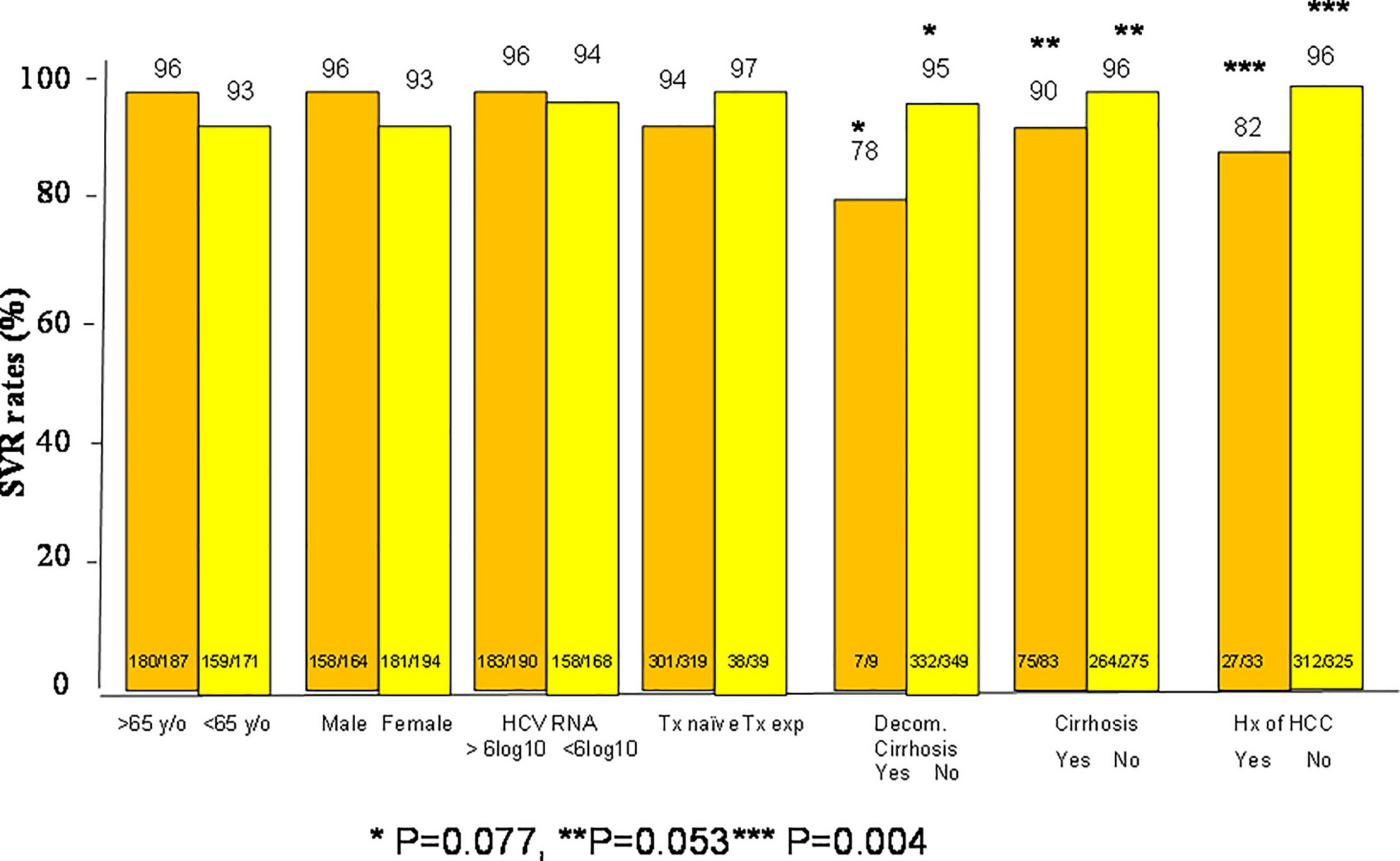
Subgroup analysis of the sustained virologocal response (SVR) rates in patients who received SOF/RBV regimen. SOF: sofosbuvir, RBV: ribavirin.

**Fig 3:**
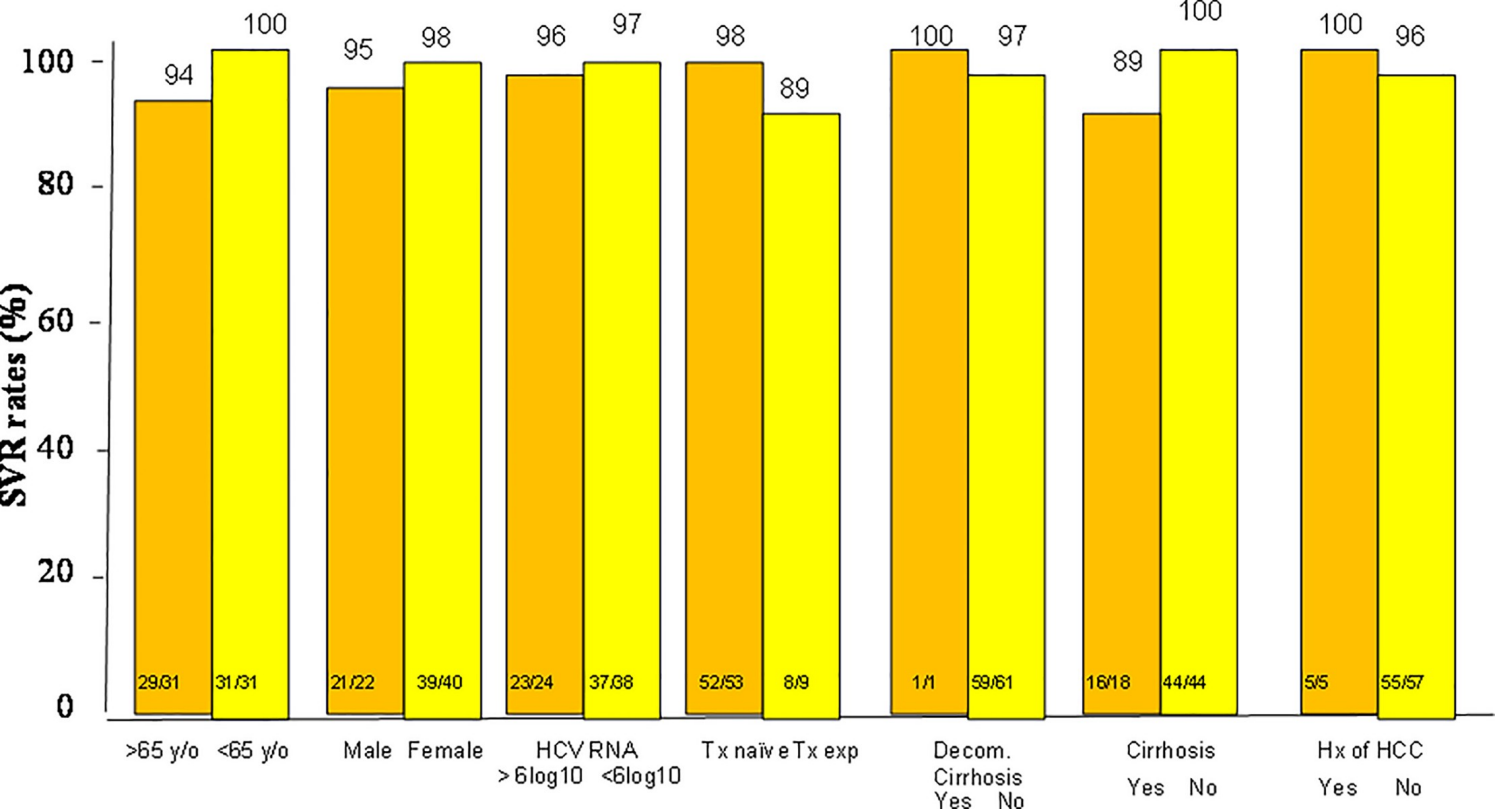
Subgroup analysis of the sustained virologocal response (SVR) rates in patients who received SOF/DCV/RBV regimen. SOF: sofosbuvir, RBV: ribavirin, DCV: daclatasvir.

**Fig 4:**
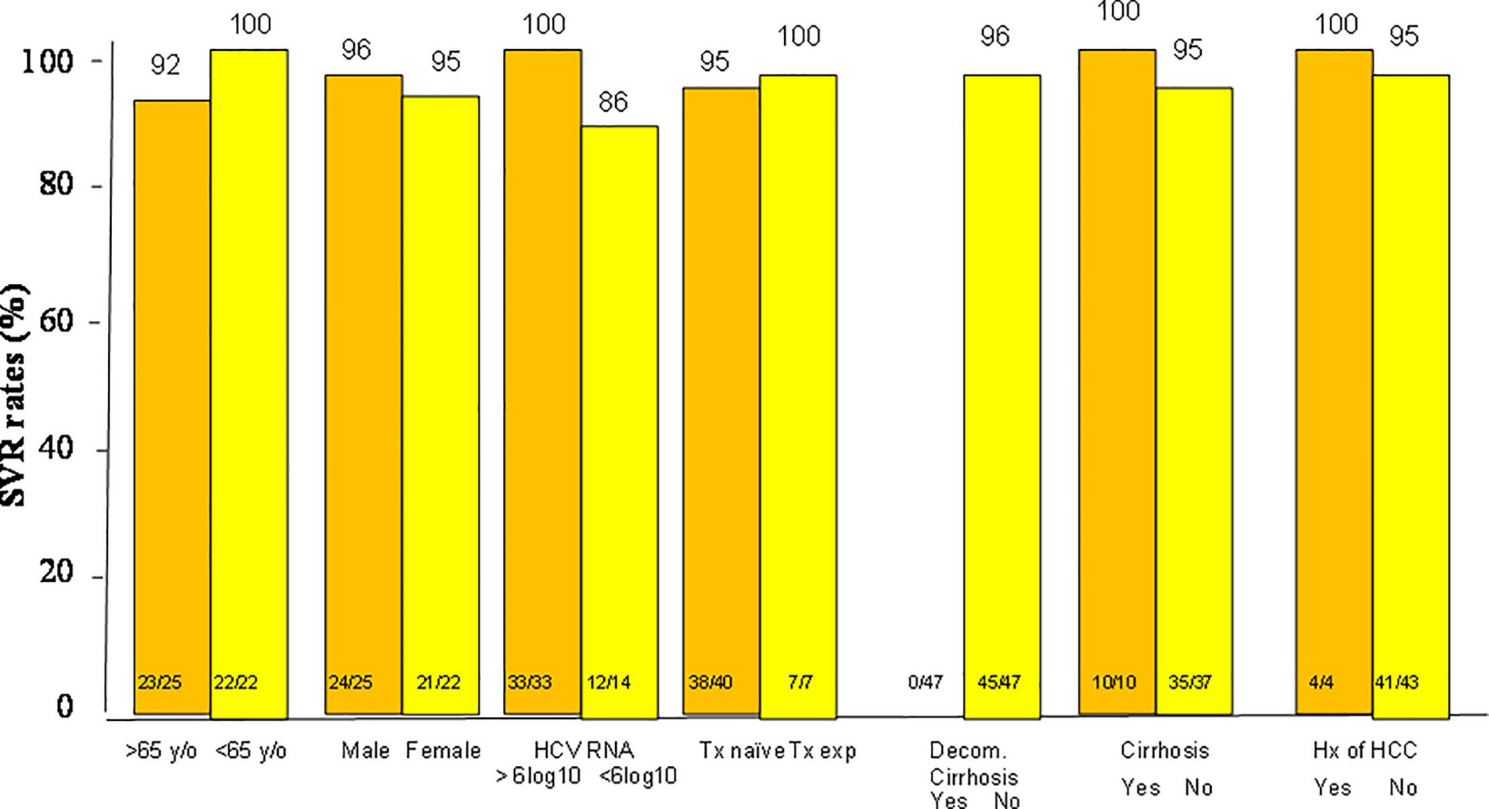
Subgroup analysis of the sustained virologocal response (SVR) rates in patients who received SOF/DCV regimen. SOF: sofosbuvir, DCV: daclatasvir.

A total of 444 of 467 (95.1%) patients achieved SVR12. In the intention to treat (ITT) population analysis, SVR12 was achieved in 339 of 359 (94.6%) patients in the SOF/RBV group and 60 of 62 (96.8%) patients in the SOF/DCV/RBV group and 45 of 47 patients (95.7%) in the SOF/DCV group (P = non-significant (NS). In the per-protocol (PP) population analysis, SVR12 was achieved in 339 of 352 (96.3%) patients in the SOF/RBV group and 60 of 61 (98.3%) patients in the SOF/DCV/RBV group and 45 of 46 patients (97.8%) in the SOF/DCV group (P = non-significant (NS) (Fig 1). Of the 467 patients who were subject to SOF-based regimens, only one patient who received SOF/RBV experienced a virological breakthrough during treatment (Table 2). Thirteen out of 352 patients (3.7%) in the SOF/RBV group, 2 out of 62 patients (3.2%) in the SOF/DCV/RBV group and 1 out of 47 patients (2.1%) in the SOF/DCV group developed virological failure. There are no differences of virological failure between the three groups of patients (P = NS). In the SOF/RBV group, a univariate analysis shows that cirrhosis and history of HCC is associated with treatment failure (P = 0.053 and 0.004 respectively) (Fig 2). Multivariate analysis shows that a history of HCC is associated with treatment failure (odds rati: 4.905, 95% confidence interval (CI): 1.321–18.205, P = 0.017) (Table 3). In the SOF/RBV/DCV and SOF/DCV group, statistical analysis does not determine any independent factors that are associated with treatment failure (Figs 3 and 4). Six patients in the SOF/RBV group and one patient in the SOF/DCV/RBV group can not complete 12 weeks of treatment but they lost follow up and do not have SVR-12 data. Adherence to DAAs as measured by medication event monitoring system or pill counts were not performed in this study. The influence of early stopping or strict adherence of DAA on SVR rate were not known from this study.

**Table 2:** Virological responses during and after treatment.

Response (HCV RNA <LLOQ)	SOF/RBV N = 358	SOF/DCV/RBV N = 62	SOF/DCV N = 47
During treatment, n/N (%)			
At week 4	339/353 (96)	60/61 (98)	45/47 (96)
At week 12	351/352 (99.7)	61/61 (100)	47/47 (100)
Post-treatment, n/N (%)			
At week 12 (SVR12)	339/358 (94.6)	60/62 (96.8)	45/47 (96)
Virological failure, n/N (%)			
During treatment	1/352 (0.3)	0	0
Relapse Early stopping (Before 12 wks)	12/352 (3.4) 6/358 (1.7)	1/62 (1.6) 1/62 (1.6)	1/47 (2.1) 0
Lost to follow up	0	0	1/47 (2.1)

SOF: sofosbuvir, RBV: ribavirin, DCV: daclatasvir

**Table 3:** Multivariable predictors of DAA treatment failure in genotype 2 chronic hepatitis C patients who received SOF/RBV regimen.

Covariate	Odds ratio	95%CI	P-value
Age	0.960	0.916–1.006	0.090
Male	0.525	0.179–1.543	0.242
Cirrhosis (Yes vs. NO)	0.658	0.166–2.616	0.552
Decompensated (Yes vs. NO)	1.119	0.127–9.885	0.919
Baseline HCC (Yes vs. NO)	4.905	1.321–18.205	0.017
Platelets (u/L)	0.990	0.978–1.001	0.080
Albumin (gm/dl)	0.506	0.122–2.094	0.347
AST (u/L)	0.999	0.980–1.019	0.945
ALT (u/L)	1.006	0.993–1.019	0.405
Bilirubin (gm/dl)	1.572	0.451–5.479	0.477

### Safety

Overall, the SOF based regimen for the treatment of genotype 2 chronic HCV is safe and well tolerated by patients (Table 4). Premature discontinuation of treatment because of adverse events is uncommon for all treatment groups. Six patients in the SOF/RBV group, 6 stopped treatment early, one refused to continue treatment but suffered no adverse effects (AE), 5 stopped treatment due to AE, 1 of whom had progression of ascites, 1 of whom had skin rash, 1 of whom had severe edema in the lower legs and an increase in creatinine level, 1 of whom experienced headache, insomnia and weakness and 1 of whom had severe itching. One patient in the SOF/RBV/DCV group refused to continue treatment but did not have any AE. One patient in the SOF/DCV group completed 12 weeks of treatment but refused to receive follow-up. No patients in the SOF/DCV group developed significant anemia, which is defined as a hemoglobin level of less than 10 mg/dl, but 10% of patients in the SOF/RBV group and 14% of patients in the SOF/RBV/DCV group developed significant anemia (P < 0.05).

**Table 4:** Adverse events.

Events, N (%)	SOF/RBV (N = 358)	SOF/DCV/RBV (N = 62)	SOF/DCV (N = 47)
SAE	4 (1.1)	2 (3)	0 (0)
AE leading to D/C	5 (1.4)	0 (0)	0 (0)
Death	0 (0)	0 (0)	0 (0)
AST, (> 5x ULN)	0 (0)	0 (0)	0 (0)
ALT, (> 5x ULN)	1 (0.3)	0 (0)	0 (0)
BIL, (> 3x ULN)	0 (0)	0 (0)	0 (0)
Cr, (> 3x ULN)	0 (0)	0 (0)	0 (0)
Decreased Hb			
(< 10 g/dl)	35 (10)*	9 (14)#	0 (0)*#
(< 8 g/dl)	3 (0.8)	2 (1.6)	0 (0)

SOF: sofosbuvir, RBV: ribavirin, DCV: daclatasvir, SAE: serious adverse events, AE: adverse events, AST: aspartate aminotransferase, ALT: Alanine transaminase, BIL: bilirubin, Cr: creatinine

Hb: hemoglobin, D/C: discontinue, ULN: upper limit of normal, g/dl: grams per deciliter.

*&#: P<0.05

## Discussion

SOF/RBV was not recommended by AASLD and EASL. However, in the APASL HCV guideline, for treatment-naive HCV GT-2 patients, daily sofosbuvir plus weight-based ribavirin for 12 weeks is recommended [6,30,31]. The data from three hospitals in Central, Southern and Eastern Taiwan show that the SOF-based regimen achieves an excellent SVR rate and has a good safety profile for genotype 2 CHC patients. Patients who receive SOF/RBV, SOF/DCV or SOF/DCV/RBV for 12 weeks all achieve very high SVR rates and were equally effective in treating genotype 2 CHC patients in the real world in Taiwan.

In a real world study for North America and Europe, Welzel et al. found that 12 or 16 weeks of SOF/RBV treatment achieves an SVR rate of 88.2% for genotype 2 CHC [17]. Another real world study involving 823 U.S. veterans with genotype-2 CHC showed that the SVR rates for SOF/RBV are 79% [32]. Another European study showed that for 236 patients with genotype 2 CHC who were treated for 12 weeks with SOF/RBV, SVR rates were achieved in only 83% of patients [15]. Several real world studies report similar results and show that SVR rates in real life were lower than those for clinical trials. However, in a phase 3b study in Taiwan, Kao et al. noted that SOF/RBV achieves a SVR rate of 100% in 83 patients with genotype 2 CHC [14]. In a recent real world study in Japan, Akahane et al. noted that treatment with SOF/RBV for 12 weeks results in SVR rates of 96.8% for a group of 914 patients who were infected with genotype II CHC s [16]. This real world study shows that treatment with SOF/RBV for 12 weeks achieves SVR rates of 94.6%, which is comparable with the real world rate for Japan and more than the real world rate for the Western world. Data for the registration clinical trials shows that the SVR rates for a SOF/RBV regimen are lower for patients with cirrhosis, especially for those who do not have previous IFN therapy [33–34]. This study finds that exposure to previous IFN treatment does not affect SVR rates and patients with cirrhosis have lower SVR rates only in a univariate analysis.

SOF/velpatasvir (VEL) combination therapy is recommended for the treatment of genotype 2 CHC patients [6,31]. In a recent study, Belperio et al. found that SVR rates do not differ between DCV + SOF (94.5%) and VEL/SOF (94.4%) or between DCV + SOF + RBV (88.1%) and VEL/SOF + RBV (89.5%) for genotype 2 CHC patients [34]. SOF/DCV with or without RBV remains an important combination regimen for genotype 2 CHC, especially in countries where SOF/VEL is not clinically approved. However, it is unclear whether adding RBV to the combination of SOF/DCV increases the SVR rate. A small Taiwanese study found that SOF/DCV with and without RBV achieves similarly high SVR rates in 32 patients with genotype 2 CHC [35]. Another small Taiwanese study notes that SOF/DCV with or without RBV results in similarly high SVR rates for 50 patients with genotype 2 CHC [36]. This study finds that the SVR rate is achieved for 60 of 62 (96.8%) patients in the SOF/DCV/RBV group and 45 of 47 patients (95.7%) in the SOF/DCV group, and adding RBV to the SOF/DCV regimen does not effect the SVR rate. Few studies compare the efficacy of SOF/RBV and SOF/DCV for the treatment of genotype 2 CHC. A recent study by Sulkowski et al. found that SOF/DCV achieves a SVR rate of 92% for 26 patients with genotype 2 CHC [20]. In a recent study, Belperio et al. found that SOF/DCV results in a SVR rate of 94.5%. [37]. In another recent study, Mangia et al. found that treatment with SOF/DCV for 12 or 24 weeks achieves a 100% SVR rate for 106 patients with genotype 2 CHC [22]. In another recent study, Swallow et al. discovered that for patients who were co-infected with genotype 1–3 CHC and HIV, the SVR12 rate is higher for patients who are treated with SOF/DCV (n = 91) than for those who are treated with SOF/RBV (n = 455) (96.7% vs 84.6%; P = 0.002) [38]. This study finds that SOF/RBV and SOF/DCV achieve similarly high SVR rates (94.6% vs. 95.5%) for the treatment of genotype 2 CHC.

In a retrospective cohort study, Prenner et al. found that 21% of patients fail to achieve SVR with HCC, compared to 12% of patients without HCC (p = 0.009) [39], but only 5% of patients were genotype 2. A recent study by Beste et al. used a cohort of US veteran patients to show that the overall SVR rate is 91.1% for non-HCC and 74.4% for HCC. The presence of HCC is associated with a lower likelihood of SVR [40]. However almost 80% of patients had genotype 1 CHC. In another recent study of 1021 patients with CHC, Yen et al. discovered that active HCC is associated with non-SVR after DAA treatment [41], but the study was heterogenous and only 32.3% of patients had genotype 2 CHC and up to eight DAA regimens were used in the study. In a recent study from Taiwan, Huang et al. in CHC patients who received DAAs treatment disclosed that a substantially but not significantly lower SVR rate, 92.1% (35/38), was observed in the patients with viable HCC compared with the SVR rate, 97.3% (72/74), in those with curative HCC (p = 0.33) [42]. But only 13% of patients in this study were genotype 2 CHC. For patients in this study who received the SOF/RBV regimens, multivariate analysis shows that a history of HCC is associated with a failure in treatment. This study focuses on patients with genotype 2 CHC who receive a SOF/RBV regimen and identifies the independent factor that is associated with a failure in treatment. Patients with genotype 2 CHC who have a history of HCC have a lower SVR rate so treatment options other than SOF/RBV may be considered.

The SOF-based regimen for the treatment of genotype 2 chronic HCV is safe and is tolerated by Taiwanese patients. Premature discontinuation of treatment because of adverse events is uncommon for all of the treatment groups but more patients in the SOF/RBV and the SOF/RBV/DCV groups develop significant anemia.

This real-world data shows that a SOF-based regimen with SOF/RBV, SOF/DCV or SOF/DCV/RB is safe and effective for the treatment of genotype 2 CHC in Taiwan. However, this study has several limitations. It does not determine the efficacy of SOF/VEL for the treatment of genotype 2 CHC because at the time of this study, SOF/VEL had not been approved in Taiwan. Recently, two real-world studies to determine the effectiveness of brand-name or generic SOF/VEL for patients with genotype 2 CHC showed that the SVR12 rates are 94% and 98%, respectively [37,43] and a combination of SOF/VEL does not result in a higher SVR rate than a SOF/DCV regimen. SOF/RBV or SOF/DCV are important regimens for the treatment of genotype 2 CHC.

In conclusion, SOF/RBV, SOF/DCV or SOF/DCV/RBV for 12 weeks achieve very high SVR rates and are equally effective in the treatment of patients with genotype 2 CHC in Taiwan. A history of HCC results in a lower SVR rate for the SOF/RBV group.
